# End-to-End Point Cloud Completion Network with Attention Mechanism

**DOI:** 10.3390/s22176439

**Published:** 2022-08-26

**Authors:** Yaqin Li, Binbin Han, Shan Zeng, Shengyong Xu, Cao Yuan

**Affiliations:** 1 School of Mathematics and Computer Science, Wuhan Polytechnic University, Wuhan 430024, China; 2 College of Engineering, Huazhong Agricultural University, Wuhan 430070, China

**Keywords:** end to end, deep learning, point cloud completion, squeeze and excitation, trilinear interpolation

## Abstract

We propose a conceptually simple, general framework and end-to-end approach to point cloud completion, entitled PCA-Net. This approach differs from the existing methods in that it does not require a “simple” network, such as multilayer perceptrons (MLPs), to generate a coarse point cloud and then a “complex” network, such as auto-encoders or transformers, to enhance local details. It can directly learn the mapping between missing and complete points, ensuring that the structure of the input missing point cloud remains unchanged while accurately predicting the complete points. This approach follows the minimalist design of U-Net. In the encoder, we encode the point clouds into point cloud blocks by iterative farthest point sampling (IFPS) and k-nearest neighbors and then extract the depth interaction features between the missing point cloud blocks by the attention mechanism. In the decoder, we introduce a new trilinear interpolation method to recover point cloud details, with the help of the coordinate space and feature space of low-resolution point clouds, and missing point cloud information. This paper also proposes a method to generate multi-view missing point cloud data using a 3D point cloud hidden point removal algorithm, so that each 3D point cloud model generates a missing point cloud through eight uniformly distributed camera poses. Experiments validate the effectiveness and superiority of PCA-Net in several challenging point cloud completion tasks, and PCA-Net also shows great versatility and robustness in real-world missing point cloud completion.

## 1. Introduction

Among the various ways to describe 3D data, point clouds are widely used for 3D data processing due to their small data size and finer rendering capabilities. Real-world point cloud data are typically acquired using laser scanners, stereo cameras, and low-cost RGB-D depth cameras. However, limitations in occlusion, light reflection, transparency of surface materials, sensor resolution, and viewing angles result in the loss of geometric and semantic information. Therefore, how to use the limited missing point cloud data to restore the original complete shape has become a hot research topic in current point cloud processing algorithms, which has important research value for point cloud 3D reconstruction and target identification.

Because 3D point clouds are unstructured and disordered, most deep learning-based methods for processing 3D data convert point clouds into sequential image collections [1] or voxel-based 3D data representations [2] in the practical task of dealing with point cloud completion. However, multiple views and voxel-based representations lead to unnecessary data redundancy and limit the output resolution. PCN [3] and FoldingNet [4] focus on learning the general features of a category rather than the local details of a specific object during the completion process and are less effective in recovering the local details of the complete point cloud. Most of the subsequent works [5,6,7,8,9] use a two-stage model, where the missing point cloud is first passed through a “simple” network, such as multilayer perceptrons (MLPs), to generate a coarse complete point cloud, and then the coarse point cloud is fed into a more “complex” network, such as auto-encoders and transformers, to enhance the local details of the complete point cloud. However, the different network complexity of the two stages results in learning more of a mapping from coarse to fine point clouds, lacking the ability to generate point clouds with fine local integrity directly from the missing ones.

**Figure 1:**
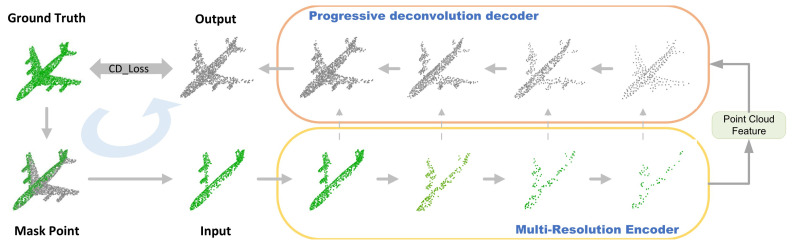
PCA-Net generates the ground-truth point cloud as a missing point cloud by the multi-view missing point cloud generation method (the viewpoints are selected as [0, 0, 1], the green part is the viewable point, and the gray part is the hidden point). Then, the green viewable points are input to multi-resolution encoder to generate point cloud feature, and the complete point cloud output is generated by progressive deconvolution decoder (the resolution of encoder is N, N/2, N/4, and N/8, and the resolution of decoder, N/8, N/4, N/2, and N). CD_Loss (Chamfer Distance in Section 3.3) to evaluate the difference between predicted and true values.

In this paper, we propose a new, conceptually simple, general framework and end-to-end approach for point cloud completion. As shown in Figure 1, this approach consists of a continuous network of encoders–decoders: a multi-resolution encoder and a progressive deconvolution decoder. In the first network framework, the complete point cloud is used to generate an “approximate” missing point cloud by the multi-view missing point cloud generation method and to form a point cloud pair with the complete point cloud as the basis for training, as shown in the mask points in Figure 1. In this process, considering previous point cloud feature extraction methods, such as Pointnet++ [10] and DGCNN [11], both first transform the point clouds into point cloud blocks using the k-nearest neighbors algorithm, and extract the features of each point cloud block by convolutional neural networks (CNNs), or customized convolutional modules. However, converting the whole point cloud into point cloud blocks is too costly, redundant, and does not consider the interaction between the point cloud blocks. We propose a simpler interactive feature fusion module, which is to use iterative farthest point sampling (IFPS) to sample key points in the complete point cloud, and then use the key points to make k-nearest neighbors to form point cloud blocks, which reduces the computational cost and the redundancy between point cloud blocks. Further, to increase the feature interaction among the point cloud blocks, we introduce the attention mechanism, which has made a big splash in both computer vision and natural language processing. Finally, considering that this interactive feature fusion module loses some feature information, we also employ the multi-resolution feature extraction to extract deeper features of the missing point cloud. The missing point clouds are mapped into potential feature vectors through this network framework, as shown in Figure 1.

In the second network framework, the potential feature vectors are fed into the progressive deconvolution decoder to predict point clouds with fine details localization and completeness, as shown in Figure 1. In this process, considering such structures as PCN [3] and MSN [7] that output the complete point cloud directly at the decoder stage, the process is complicated, and it is difficult to recover the local details of the complete point cloud. For example, the basic shape of the chair can be recovered, but the connection between the chair legs will be ignored. We adopted a progressive point cloud generation method that can predict point clouds with different resolutions from layers of different depths. However, in the process of progressive upsampling, the classical linear interpolation and bilinear interpolation are both non-learning interpolation methods, which cannot adapt to different classes of 3D models. Both only use the point cloud of the previous resolution for the interpolation, completely ignoring the information that already exists in the missing point cloud at that resolution. Therefore, inspired by a PDGN [12], we propose a learning-based trilinear interpolation method, which can simultaneously use the coordinate space, feature space, and neighborhood information of each point in the missing point cloud of the same resolution to generate fine-grained missing regions.

We finally compare the predicted complete point cloud with the real complete point cloud from the training process by multi-level CD loss to guide the prediction of our method. After the prediction model is trained, the missing point clouds are fed into the model to predict the complete aircraft point clouds. We experimented with the method on the Modelnet40 dataset [13] and ShapeNet-Part dataset [14], and both achieved excellent performance. In addition, that the current PCN [3] and Vrc-Net [9] datasets are complicated to produce and can only be applied to supervised learning relatively singularly was taken into consideration. Accordingly, we proposed a simple and efficient multi-viewpoint missing point cloud generation method using a 3D point cloud hidden point removal algorithm [15] and generated eight viewpoints of missing point clouds for each complete 3D point cloud model in the Modelnet40 dataset and ShapeNet-Part dataset, which provides a database for subsequent in-depth research. The main contributions of PCA-Net can be summarized as follows:

- We propose a new end-to-end approach to the point cloud completion network framework.
- We propose an interactive feature fusion module that uses an attention mechanism to increase the interactive fusion of features between each point cloud block.
- We develop a new progressive inverse folded product network that uses learning-based trilinear interpolation to generate complete point clouds with fine detail localization.
- This paper also proposes a simple and efficient multi-view missing point cloud generation method, which provides a database for subsequent in-depth research.

The rest of this paper is organized as follows. Section 2 describes the related work. In Section 3, we present the asymptotic end-to-end point cloud completion model. Section 4 presents the multi-view missing point cloud datasets generation. Section 5 presents the experimental results, and Section 6 presents the conclusions.

## 2. Related Work

Our work builds on prior work in several domains: point-based deep learning, attention mechanism, and point cloud completion.

**Point-based deep learning**. The current research on 3D deep learning in the field of point clouds is divided into two main approaches. One is to transform 3D point clouds into regular structured data and then use the currently available deep learning methods to process them. The main ones are to transform 3D objects into a collection of 2D views [16], which can be processed using CNNs, transformers, etc. However, this approach increases the computational effort and lacks a 3D view. There is also the voxelization of 3D objects [17], but this approach leads to a heavy memory burden and high computational complexity. Another approach is to construct special operations suitable for 3D unstructured geometric data for 3D deep learning. PointNet [18] was the first to directly combine deep learning with 3D point clouds. Subsequently, PointNet++ [10] was proposed to group and layer point clouds and use PointNet to capture the local and global information of point clouds. Point-GNN [19] combines 3D point clouds with graphical neural networks (GNNs), which are widely used in 2D, and achieves good results. PointCNN [20] proposes a convolution operation on irregular point cloud data by X-transform. EdgeConv [11] proposes an EdgeConv operator that can learn point cloud features by local topology. Recent work has shown a very competitive and compelling performance on standard datasets. For example, the state-of-the-art methods SpecGCN [21], SpiderCNN [22], DGCNN [18], and PointCNN [20] achieve perfect accuracy for object classification tasks on the ModelNet40 dataset.

**Attention mechanism**. The attention mechanism aims to mimic the human visual system by focusing attention on features relevant to the target rather than on the whole scene containing some irrelevant background. For image-related tasks, attentional maps can be generated based on spatial [23] or channel-related information [24], while some approaches combine both for better information integration. Currently, there is a corresponding integration of attention mechanisms with many domains as well. Swin Transformer [25] uses computation by shifting windows in image feature processing, which allows self-attentive computation to be limited and brings higher efficiency and accuracy. There is also SwinFusion [26] which combines self-attentive intra-domain fusion units and cross-attentive-based inter-domain fusion units to mine and integrate long dependencies within and across the same domain. In addition, point cloud processing tends to utilize self-attentive structures, which estimate random dependencies without considering a specific order between elements. Among them, PointASNL [27] uses the self-attention mechanism to obtain finer local point group features, and PCT [28] applies transformers to process point cloud features to enhance the characterization of point cloud features. It also achieves a perfect performance in the areas of the classification, segmentation, and completion of 3D point clouds.

**Point cloud completion**. The in-depth study of deep learning applied to 3D point clouds has gradually transformed the point cloud completion task to one where the shape of a complete 3D point cloud can be recovered using a partial point cloud as input. Among them, a PCN [3] maps the global features learned from a partial input point cloud to a coarse complete point cloud and restores it with secondary refinement by a folding decoder. TopNet [5] proposes to predict the complete point cloud shape using a tree structure decoder. AlasNet [6] further represents the 3D shape as a collection of surface parameters and generates more complex shapes by a 2D mesh to the 3D surface element. MSN [7] predicts a complete but coarse-grained point cloud, a set of parametric surface elements, through a linear folding generation method as the first stage. Then, in the second stage, the coarse-grained predicted point cloud is merged with the input point cloud by a novel sampling algorithm to generate a fine-grained point cloud. GRNet [8] converts the point cloud into an equally spaced voxel grid, extracts features in the grid using a 3D convolutional layer, and inputs the extracted 3D feature vectors into a demeshing layer to generate the predicted point cloud. Regarding PF-Net [29], in order to maintain the original partial spatial arrangement, a point cloud fractal network for repairing missing point clouds is proposed, which takes partial point clouds as input and only outputs the missing part of the point cloud instead of the whole object. VrcNet [9] proposes a variational framework network to repair missing point clouds by learning feature information of the complete point cloud in the auto-encoder and optimizes the network by another point cloud enhancement of the local details.

## 3. Method

**Figure 2:**
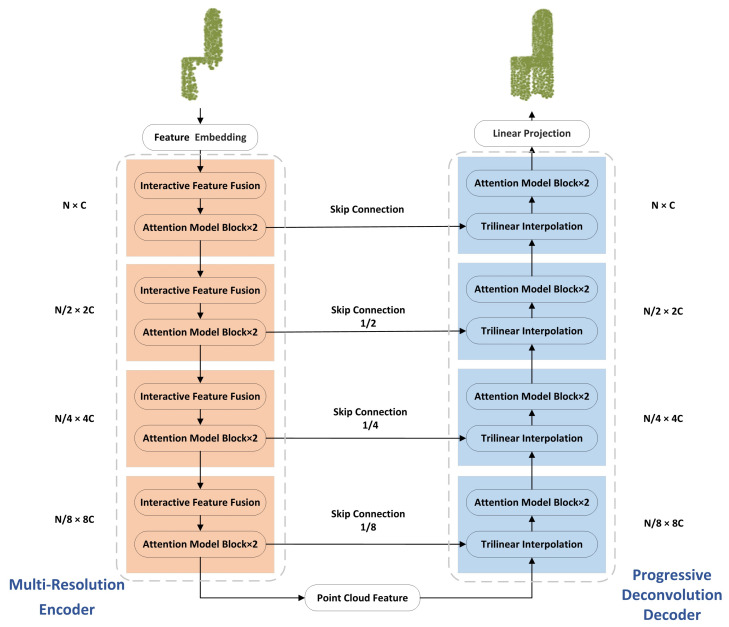
Network framework of PCA-Net. The input to PCA-Net is a missing point cloud, and the point cloud features are extracted using multi-resolution encoder (Section 3.1), including interactive feature fusion (Section 3.1.1) and attention model (Section 3.1.2). The extracted point cloud feature is used to generate high-quality complete point clouds by progressive deconvolution decoder (Section 3.2), including trilinear interpolation (Section 3.2.1). Linear projection represents features to generate complete point clouds by MLPs. N, 2/N, 4/N, 8/N and C, 2C, 4C, 8C indicate the resolution and feature dimension of the point cloud under that level, respectively.

This section focuses on the PCA-Net network framework, whose network structure is shown in Figure 2. The input of this network is a partial point cloud, and the output is a complete shaped point cloud. The overall architecture consists of two basic building blocks, multi-resolution encoder and progressive deconvolution decoder.

### 3.1. Multi-Resolution Encoder

The architecture of the multi-resolution encoder is shown in Figure 2. The goal is to encode the input missing point cloud into a new high-dimensional feature space that serves as the basis for the point cloud completion task. The encoder first embeds the input coordinates into a new feature space by feature embedding (convolutional residual, normalization, and activation function layers). The interactive features between the points are obtained by the interactive feature fusion module proposed in Section 3.1.1. Whereas effective feature fusion is the core of multi-scale feature representation, a straightforward approach is to join multi-scale features and then perform convolution operations simply. However, this simple approach cannot capture the dependencies and global interactions between features at different scales. Therefore, this paper uses the attention mechanism to achieve efficient interactions between multi-scale features. These multi-scale features contain local, global, and low-level and high-level features, which can enhance the ability of the network to extract semantic and geometric information. In this paper, the number of output point clouds in the four stages are N, N/2, N/4, and N/8; the dimensions are C, 2C, 4C, and 8C; and two single-level self-attention operations are performed in each stage to keep the computational complexity within an acceptable range.

#### 3.1.1. Interactive Feature Fusion

**Figure 3:**
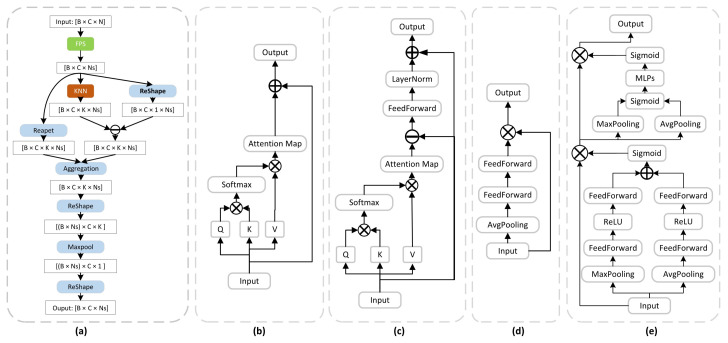
(**a**) Interactive Feature Fusion. (**b**) Point-Attention. (**c**) Offset-Attention. (**d**) Squeeze and Excitation (SE) block. (**e**) Convolutional Block Attention Module (CBAM).

This paper designs a point cloud interactive feature fusion strategy to enhance local feature extraction by means of neighborhood embedding. As shown in Figure 3a, interactive feature fusion consists of iterative farthest point sampling (IFPS) and neighborhood feature aggregation. In the next aggregation process, k-nearest neighbors is used to expand the perceptual domain. In the point cloud sampling process, the neighborhood feature aggregation fuses the local neighborhood features of each point in the k-nearest neighbors search grouping. Take the *i*-th point as example; the feature vector of its current feature space is $f_{i}$. Then, use the iterative farthest point sampling (IFPS) sample to $f_{s}$, with $f_{s}$ as the key point through the k-nearest neighbors in the current feature space are denoted by $\left\{f_{s1},\cdots ,f_{sk}\right\}$. The neighborhood of the current feature space is calculated operation to obtain a high-level neighborhood feature set $\left\{h_{s1},\cdots ,h_{sk}\right\}$. The calculation method of $h_{sk}$ in the manner of Equation (1):

(1) $$h_{sk}=MLP\;\left[f_{s}U\left(f_{s}-f_{sk}\right)\right],f_{sk}\in \left\{f_{s1},\cdots ,f_{sk}\right\},h_{sk}\in \left\{h_{s1},\cdots ,h_{sk}\right\}$$

where *MLP*[· ] represents a multilayer perception operation with shared parameters.

#### 3.1.2. Attention Mechanism

Qiu [30] used and compared various types of attentional mechanisms for various tasks in 3D point clouds. These involve the 2D attention squeeze and excitation (SE) block [24], shown in Figure 3d; the convolutional block attention module (CBAM) [31], shown in Figure 3e; and the 3D attention point-attention [32], shown in Figure 3b, and offset-attention [28], shown in Figure 3c.

The squeeze and excitation (SE) [24] module represents channel attention and can adaptively learn the inter-dependencies between different channels. First, the feature map is global average pooling to obtain the global features of each channel in the current feature map. Then, the dependencies between each channel are obtained by two fully connected MLP layers and this dependency is converted into weights by a sigmoid activation function, and finally, the weighted feature map is used as the input to the next layer of the network. The convolutional block attention module (CBAM) [31] consists of a channel attention module and a spatial attention module. The channel attention module first applies global max pooling and global average pooling at each channel of the feature map to obtain the global features of the two feature maps, and then feeds them into the shared MLP layer separately to obtain the dependency between each channel, and finally converts this dependency into weights by summing the two through the activation function. Finally, the weighted feature maps are used as the input of the spatial attention module. The spatial attention module performs global max pooling and global average pooling for each channel of the feature map to obtain the global features of the two feature maps and splice them by channel. Then, the spatial relationship within each channel is obtained after the MLP layer, and the spatial relationship is converted into weights by the activation function. Finally, the weighted feature maps are used as the input of the next layer of the network. That is, given the aggregated features as $F_{0}$ inputs, $SE\left(F_{0}\right)$ is calculated by Equation (2); CAM$\left(F_{0}\right)$ is calculated by Equation (3); SAM$\left(F_{0}\right)$ is calculated by Equation (4); CBAM$\left(F_{0}\right)$ is calculated by Equation (5).

(2) $$SE\left(F_{0}\right)=F_{0}\cdot \sigma \left(MLP\left(MLP\left(AvgPool\left(F_{0}\right)\right)\right)\right)$$

(3) $$\mathrm{CAM}\left(F_{0}\right)=\sigma \left(\mathrm{MLP}\left(\sigma \left(\mathrm{MLP}\left(\mathrm{AvgPool}\left(F_{0}\right)\right)\right)\right)+\mathrm{MLP}\left(\sigma \left(\mathrm{MLP}\left(\mathrm{MaxPool}\left(F_{0}\right)\right)\right)\right)\right)$$

(4) $$\mathrm{SAM}\left(F_{0}\right)=\sigma \left(\mathrm{MLP}\left(\left[\mathrm{MaxPool}\left(F_{0}\right);\mathrm{AvgPool}\left(F_{0}\right)\right]\right)\right)$$

(5) $$\mathrm{CBAM}\left(F_{0}\right)=F_{0}\cdot \sigma \left(\mathrm{MLP}\left(\left[\mathrm{MaxPool}\left(CAM\left(F_{0}\right)\right);\mathrm{AvgPool}\left(\mathrm{CAM}\left(F_{0}\right)\right)\right]\right)\right)$$

where σ denotes the activation function and [] denotes the vector splicing.

The point-attention [32] module follows the basic structure of self-attention, where *Q*, *K*, and *V* are the query, key, and value matrices, respectively, generated by making a linear variation of the input feature map. The weights of attention are computed through the query and key matrices and the weights of attention are weighted with the value matrices. Finally, the relationship between input and output is enhanced by skipping connections. The offset-attention [32] differs from the point-attention module in using a self-attention structure to estimate the offset between the input features and the attention features to enhance the interaction between the feature maps. That is, given aggregated features $F_{0}$ as inputs, $PA\left(F_{0}\right)$ is calculated by Equation (7); $OA\left(F_{0}\right)$ is calculated by Equation (8).

(6) $$\left(Q,K,V\right)=F_{0}\cdot \left(W_{q},W_{k},W_{v}\right)$$

(7) $$PA\left(F_{0}\right)=\mathrm{softmax}\left(Q\cdot K^{T}\right)\cdot V+F_{0}$$

(8) $$OA\left(F_{0}\right)=MLP\left(\mathrm{softmax}\left(Q\cdot K^{T}\right)\cdot V-F_{0}\right)+F_{0}$$

where $W_{q}$, $W_{k}$, $W_{v}$ denotes the shared learnable linear transformation.

In this paper, we embed various types of attention into the network and experimentally verified that the squeeze and excitation (SE) block [24] has superior performance in multi-scale feature fusion in Section 5.3.

### 3.2. Progressive Deconvolution Decoder

The decoder takes feature vectors as input and aims to output complete 3D point cloud shapes. The baseline of the progressive deconvolution decoder is the learning-based trilinear interpolation proposed in Section 3.2.1. Based on FPN [33] and PDGN [12], a progressive method of full point cloud generation is proposed to generate full point cloud from low-resolution to high-resolution step by step and predict primary, secondary, and detailed points from layers with different feature depths. The primary and secondary points are matched with the corresponding feature points, and the number of points is gradually increased, and a learning-based trilinear interpolation approach generates their high-dimensional feature maps. The attention mechanism is used to process the high-dimensional feature maps, and the MLPs are used to generate the 3D coordinates of the point clouds at each resolution to propagate the overall geometric information to the final detailed points. Throughout the point cloud completion process, the output point cloud resolutions of the four stages are N/8, N/4, N/2, and N; the dimensions are 8C, 4C, 2C, and C.

#### 3.2.1. Learning-Based Trilinear Interpolation

**Figure 4:**
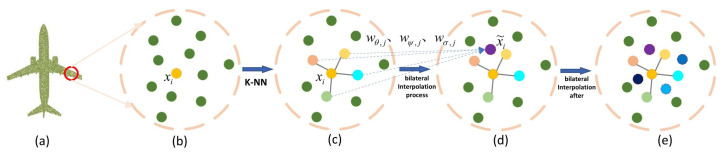
The operation process of learning-based trilinear interpolation. First, the similarity between pairs of points in the feature space (**b**) is defined from the complete point cloud (**a**). The k-nearest neighbors (k-NN) with defined similarity are selected in the feature space (**c**). Then, interpolation is performed in the neighborhood, and its interpolation process is shown in (**c**,**d**). Finally, a zoomed feature map is formed (**e**).

The irregularity of the point cloud makes it impossible to interpolate the feature map directly. However, in 2D images [34] and 3D point clouds [10,11,18,20,28], many of them are used to achieve the desired task by a neighborhood. In this paper, the neighborhood of each point in the feature space is constructed using k-nearest neighbors. As shown in Figure 4c, the k-nearest neighbors with similarity definition can be selected in the feature space. The decoder is defined as progressive generation, the k values are set to 4, 8, 16, and 32, corresponding to four different resolutions, respectively.

The classical linear interpolation and bilinear interpolation methods are non-learning interpolation methods, which cannot utilize the neighborhood information of each point in space. This paper proposes a learning-based trilinear interpolation method to generate a high-resolution complete point cloud feature map using the spatial coordinates of each point, the neighborhood features, and the missing point cloud features at the same resolution. Take the *i*-th point as example; its original *XYZ* space coordinate is represented by $p_{i}$, and the feature vector of its current feature space is $x_{i}$. The k-nearest neighbors searched in the space are defined as a set $\left\{p_{i1},\cdots ,p_{\mathrm{iK}}\right\}$, while the k-nearest neighbors in the current feature space are denoted by $\left\{x_{i1},\cdots ,x_{\mathrm{iK}}\right\}$. Assuming the feature vector of the missing point cloud at the same resolution as $p_{i}$ is $y_{i}$, while the k-nearest neighbors in the current feature space are denoted by $\left\{y_{i1},\cdots ,y_{\mathrm{iK}}\right\}$.

The features of its local coordinate space are denoted by $\phi \left(p_{i},p_{j}\right)$ as Equation (9):

(9) $$\phi \left(p_{i},p_{j}\right)=\mathrm{ReLU}\;\left(W_{\theta ,j}^{⊤}\left(p_{i}-p_{j}\right)\right),\;p_{j}\in \left\{p_{i1},\cdots ,p_{\mathrm{iK}}\right\}$$

the features of its local feature space are represented by $\gamma \left(x_{i},x_{j}\right)$ as Equation (10):

(10) $$\gamma \left(x_{i},x_{j}\right)=\mathrm{ReLU}\;\left(W_{\psi ,j}^{⊤}\left(x_{i}-x_{j}\right)\right),\;x_{j}\in \left\{x_{i1},\cdots ,x_{\mathrm{iK}}\right\}$$

the spatial features of the local features of the missing point cloud under the same resolution are characterized by $\omega \left(y_{i},y_{j}\right)$. The calculation process is as in Equation (11):

(11) $$\omega \left(y_{i},y_{j}\right)=\mathrm{ReLU}\;\left(W_{\sigma_{j}}^{⊤}\left(y_{i}-y_{j}\right)\right),\;y_{j}\in \left\{y_{i1},\cdots ,y_{\mathrm{iK}}\right\}$$

where ReLU is the activation function, $W_{\theta ,j}^{⊤}$, $W_{\psi ,j}^{⊤}$, $W_{\sigma_{j}}^{⊤}$ indicates weights to be learned.

We formulate the trilinear interpolation as Equation (12):

(12) $${\tilde{x}}_{i,1}=\frac{\sum_{j=1}^{k}\phi_{1}\left(p_{i},p_{j}\right)\gamma_{l}\left(x_{i},x_{j}\right)\omega_{1}\left(y_{i},y_{j}\right)x_{j,1}}{\sum_{j=1}^{k}\phi_{1}\left(p_{i},p_{j}\right)\gamma_{l}\left(x_{i},x_{j}\right)\omega_{1}\left(y_{i},y_{j}\right)}$$

${\tilde{x}}_{i,1}$ is the *l*-th element of the interpolated feature ${\tilde{x}}_{i}$, k denotes the number of k-nearest neighbors.

As shown in Figure 4c,d, the new interpolated feature ${\tilde{x}}_{i}$ can be obtained from the neighborhood of $x_{i}$ with the trilinear weight. For each point, we perform the trilinear interpolation in the k-nearest neighbors to generate new k points. Therefore, we can obtain a high-resolution feature map, where the neighborhood of each point contains 2k points. After the interpolation, the attention mechanism is applied to the amplified feature mapping and used as the output of the inverse convolution network.

### 3.3. Loss Function

The loss measure in the point cloud completion process represents the difference between the true complete point cloud corresponding to the missing point cloud and the predicted point cloud. Fan [35] proposed two alignment-invariant metrics to compare the difference between disordered point clouds, namely Chamfer Distance (CD) and Bulldozer Distance (EMD). Because the Bulldozer Distance (EMD) occupies more memory and takes longer to calculate, while the Chamfer Distance (CD) is more efficient to calculate, this paper chooses the Chamfer Distance as the loss function for point cloud completion as follows Equation (13):

(13) $$L_{\mathrm{CD}}\left(S_{1},\;S_{2}\right)=\frac{1}{2}\left(\frac{1}{\left|\;S_{1}\right|}\sum_{x\in S_{1}}\min_{y\in S_{2}}∥x-y∥+\frac{1}{\left|\;S_{2}\right|}\sum_{y\in S_{2}}\min_{x\in S_{1}}∥x-y∥\right)$$

The mean nearest square distance, referred to as the Chamfer Distance (CD), between the predicted point cloud $S_{1}$ and the true point cloud $\;S_{2}$ is measured in Equation (13) above. The progressive deconvolution completion network is a special progressive deconvolution 3D point cloud completion network in which the complete point cloud is generated in four stages with resolutions. The predicted point cloud outputs of the four stages are denoted by $Y_{1}$, $Y_{2}$, $Y_{3}$, and $Y_{4}$, the true complete point clouds sampled from the true point cloud by IFPS to N/8, N/4, N/2, and N resolutions are denoted by $Y_{\mathrm{gt}}$, $Y_{\mathrm{gt}}^{\prime }$, $Y_{\mathrm{gt}}^{\prime \prime }$, and $Y_{\mathrm{gt}}^{\prime \prime \prime }$. The Chamfer Distance (CD) of the four stages is denoted by $d_{{\mathrm{CD}}_{1}}$, $d_{{\mathrm{CD}}_{2}}$, $d_{{\mathrm{CD}}_{3}}$, and $d_{{\mathrm{CD}}_{4}}$. The complete loss function for the training process is shown in Equation (14):

(14) $$L_{\mathrm{com}\;}=d_{{\mathrm{CD}}_{1}}\left(Y_{1},Y_{\mathrm{gt}}\right)+d_{{\mathrm{CD}}_{2}}\left(Y_{2},Y_{\mathrm{gt}}^{\prime }\right)+d_{{\mathrm{CD}}_{3}}\left(Y_{3},Y_{\mathrm{gt}}^{\prime \prime }\right)+d_{{\mathrm{CD}}_{4}}\left(Y_{4},Y_{\mathrm{gt}}^{\prime \prime \prime }\right)$$

## 4. Multi-View Missing Point Cloud Datasets Generation

The amount of data is crucial in the training of deep learning, but it is difficult to obtain such paired data. Both a PCN [3] and PF-Net [29] use 3D mapping software to draw some common objects in reality and generate missing point clouds by some missing methods. Among them, the missing point clouds generated in PF-Net [29] are different from the missing point clouds in daily depth cameras and LiDAR, which do not reach a good completion effect. In addition, the PCN [3] artificially uses third-party software to guide the generation of missing point clouds. Although the point clouds generated by this method are extremely similar to the missing point clouds encountered in daily life, the production process is more complicated. Another great drawback is that the paired data generated by various methods can only be applied to single supervised learning but are less applicable to the current research in the self-supervised and unsupervised fields. Sagi [15] proposes a method for 3D point cloud hidden point removal; given only one viewpoint, the visible points in the 3D point cloud are controlled by adjusting a visibility threshold. A larger threshold R indicates that more points are visible points. Based on this paper, a simpler and more efficient method of generating partial point clouds based on 3D point clouds with multiple viewpoints is designed, and R = 100 is chosen, but the resolution of the generated partial point clouds exists differently.

**Figure 5:**
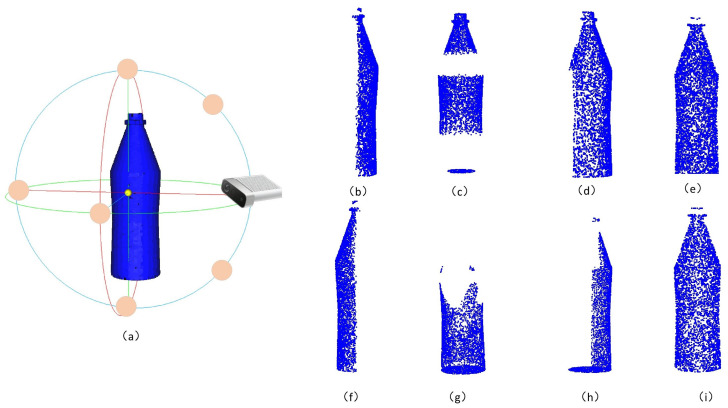
Figure (**a**) shows the camera poses around the complete 3D point cloud. Figure (**b**–**i**) represent the partial point cloud generated by different camera poses.

The 3D point cloud of the ShapeNet-Part dataset and Modelnet40 dataset is taken as the experimental object. The missing point cloud is generated by the method, which forms a data pair with the complete point clouds to form the training dataset. Borrowing from the generation of the datasets in the PCN, the following Figure 5a shows the camera pose map. Each orange circle indicates a camera pose, where the relative poses between the eight camera poses are fixed, but each training camera pose is randomly selected, and Figure 5b–i show the missing point clouds generated by the eight camera poses, respectively.

Compared with previous missing point cloud datasets, PCN [3], PF-Net [29], and VrcNet [9] have the following advantages: 1. The object is a 3D point cloud that can be embedded in the network without additional storage space, and the experiment is fast and convenient. 2. Using a uniformly distributed camera view, the number of camera poses can be adjusted according to demand, and the ability to generate complete 3D shapes under partial conditions can be better evaluated by using fewer complete shapes during training. 3. The number of missing and complete point cloud pairs can be arbitrarily increased to generate corresponding missing point clouds for complete point clouds of different resolutions. 4. In addition, 3D shape completion methods can also be used for other missing point cloud tasks, such as classification, alignment, key point extraction, and some new self-supervised domain learning.

## 5. Experiments

**Experimental datasets**. The ModelNet40 dataset [13] contains 12311 CAD models in 40 object classes and is widely used for point cloud shape classification and surface normal estimation benchmarking. The standard 9843 objects were used for training and 2468 objects for evaluation. The ShapeNet-Part dataset [14] contains 13 different objects with a total number of 14,473 shapes (11,705 for training and 2768 for testing). All input point cloud data are centered at the origin and the coordinates are normalized to [−1, 1]. The training point cloud data were created by sampling 2048 points per object FPS.

**Evaluation metrics**. The proposed method is evaluated by calculating the Chamfer Distance (CD) between the predicted complete shape and the true complete shape. Considering the sensitivity of the CD to the outliers, the distance between the object surfaces is evaluated using the reconciled mean between the accuracy of the F-score and the chamfering rate.

**Implementation details**. The method proposed in this paper is implemented on PyTorch, all modules are trained alternatively by the ADAM optimizer with an initial learning rate of 0.0001, a batch size of 16, and are trained using a Tesla P100 GPU. The batch normalization (BN) and RELU activation units are used in the encoder and the adjacent points are set at different resolutions of 2048. In the decoder, the output size of the different resolution deconvolution networks is 256, 512, 1024, and 2048, respectively. Mapping from high-dimensional features to point cloud coordinates is achieved using the MLP to generate the coordinates of the point clouds. The Tanh activation function is used after the MLP is completed. Each network was trained 100 times separately.

### 5.1. Unsupervised Point Cloud Completion Results

**Quantitative evaluation**. The missing point cloud generation method introduced in Section 4 is used, and the datasets are generated from 40 classes of high-quality 3D point clouds in ModelNet to generate missing and complete point cloud pairs. These point cloud pairs are divided into a training set (9843 shape pairs) and a test set (2468 shape pairs), and none of the point cloud pairs in the test set are included in the training set. The training process divides the ModelNet dataset [13] into ModelNet10 (containing ten categories) and ModelNet40 (containing 40 categories). Validating the experimental effect of the method in this paper can better evaluate the ability of the method in this paper to generate complete shapes under the missing condition. The same training strategy was used to train all the methods on each of the datasets. The CD loss and F-scores of all the evaluated methods trained on the ModelNet10 dataset are shown in Table 1 and Table 2, respectively. The table also shows each class’s CD loss and the F-scores values for comparison. The CD loss and F-scores values of all the evaluated methods under ModelNet40 dataset training are shown uniformly in Table 3. The methods in this paper outperform the existing competing methods in terms of both the CD loss and F-scores values.

To validate the applicability of the methods in this paper on other datasets, validation was performed on the benchmark dataset ShapeNet-Part [14], where it was trained using the same training strategy as the previous datasets. The CD losses of all the evaluated methods are shown in Table 4. The experiments validate that the method in this paper outperforms the existing competing methods.

**Figure 6:**
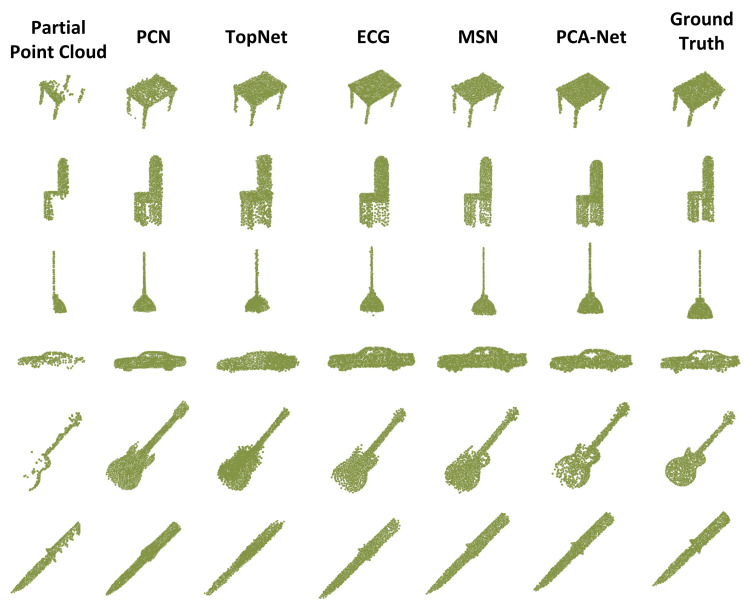
Qualitative completion results (2048 points) for the ShapeNet-Part dataset using different methods. PCA-Net generates better complete point clouds than other methods.

**Qualitative evaluation**. The results of the qualitative comparison are shown in Figure 6. Compared with other methods, PCA-Net keeps the input missing point cloud unchanged while recovering the missing structure. For example, for the missing legs of the chair and table (the first and second rows in Figure 6), we can not only predict the location of the missing legs accurately but also make the recovered shape more uniform. In the third row of Figure 6, we reconstruct the complete chandelier from one-half of the chandelier. The other methods ignore the interface between the lamp cord and the shade, but our approach retains this detail of the complete chandelier. In the missing car point cloud in the fourth row of Figure 6, most other methods only recover the shape of the car and the tires, while we recover the more complex cab shape of the car. In the fifth row of the guitar point cloud in Figure 6, the missing part of the point cloud makes the completion more difficult, but we can still generate a relatively complete and fine completion result. Therefore, PCA-Net can effectively reconstruct the complete shape when dealing with some more complex structures.

### 5.2. Supervised Point Cloud Completion Results

The above experiments are a self-supervised learning–training process, while the actual point cloud completion 3D benchmark is a supervised learning process, to evaluate the point cloud completion capability of PCA-Net on supervised learning, after generating the missing point clouds on the ModelNet10 dataset (using the method in Section 4) normalized to [−1, 1] and then forming point cloud pairs with the complete point clouds. The experimental process is trained in the same way as the 3D benchmark for the point cloud completion. PCA-Net and other methods are given in the following Table 5, and the comparison of the results of the CD loss and F-scores values shows that PCA-Net outperforms the other compared methods.

### 5.3. Qualitative Evaluation of PCA-Net Network

**Analysis of attentional mechanisms**. To demonstrate the effectiveness of the attention module for the efficient interaction between the multi-scale features, PointCNN [20] is used as the baseline model. We trained PointCNN [20], PCA-Point (attention using point-attention), PCA-CBAM (attention using convolutional block attention module), PCA-Offset (attention using offset-attention), and PCA-SE (attention using squeeze and excitation), and the overall classification accuracy was evaluated. The experimental results are shown in Table 6, and PCA-SE shows the best performance.

**Analysis of skip connection settings in the decoder**. In this paper, we try to use different methods as decoders to generate complete point clouds and verify the necessity of skipping connections in decoders by two methods: learning-based bilateral interpolation and learning-based trilinear interpolation. Validating the network improvements on the ModelNet10 dataset, as can be seen from Table 7, the learning-based trilinear interpolation method has improved the completion results compared to the learning-based bilateral interpolation method.

### 5.4. Shape Completion on Real-World Partial Scans

**Figure 7:**
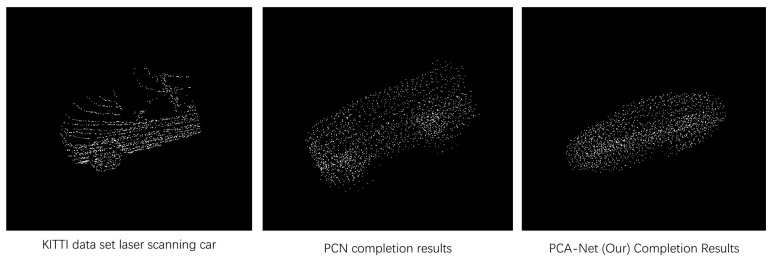
LIDAR-captured car point clouds (missing point clouds) in the KITTI dataset, with completion results on PCN [3] and PCA-Net.

To further evaluate PCA-Net, validation was performed using partial car data from the KITTI dataset [37], which uses LIDAR-captured point clouds. The method is trained on the ShapeNet-Part dataset [14] to complete the sparse LiDAR data in the KITTI dataset [37], and the qualitative completion results are shown in Figure 7, where the target point cloud generated by PCA-Net is complete and smooth compared to the PCN [3].

## 6. Conclusions

In this paper, we propose an end-to-end point cloud completion method based on the U-Net shape fusion attention mechanism and trilinear interpolation, referred to as PCA-Net. The method is able to generate a complete point cloud of the target with rich semantic contours and detailed features while preserving existing missing point cloud contours. When the training dataset is large enough, there is an opportunity to repair any complex random missing point cloud. Moreover, the large-scale datasets-based production method presented in this paper is applicable to any 3D point cloud data. In summary, the in-depth application of the method can greatly improve the accuracy of 3D recognition and bring new possibilities for the research of self-driving cars and 3D reconstruction.

## Figures and Tables

**Table 1:** Shape completion results (CD $\times \;10^{3}$) on ModelNet10 point cloud dataset (2048 points).

Category	PCN [3]	Top-Net [5]	ECG [36]	MSN [7]	Vrc-Net [9]	PCA-Net (Ours)
Bathtub	7.652	7.754	5.391	**3.008**	6.863	4.199
Bed	3.383	3.590	2.190	2.119	4.077	**2.079**
Chair	4.790	6.304	3.935	3.431	4.526	**3.293**
Table	5.286	5.384	4.137	3.766	4.693	**3.170**
Desk	10.839	10.889	12.346	6.470	7.230	**6.451**
Dresser	4.055	4.671	6.445	**2.349**	6.797	2.721
Sofa	3.549	3.783	2.165	**2.094**	4.808	2.594
Monitor	3.848	4.852	3.450	3.527	4.402	**3.189**
Night-stand	4.986	7.211	12.029	**3.885**	6.556	4.771
Toilet	3.848	5.893	4.458	3.297	5.138	**3.125**
Mean	4.334	5.433	3.988	2.778	2.982	**2.325**

The number in bold denotes it is the best result.

**Table 2:** Shape completion results (F−Score@1%) on ModelNet10 point cloud dataset (2048 points).

Category	PCN [3]	Top-Net [5]	ECG [36]	MSN [7]	Vrc-Net [9]	PCA-Net (Ours)
Bathtub	0.15	0.14	0.35	**0.70**	0.20	0.56
Bed	0.33	0.24	0.36	0.43	0.32	**0.49**
Chair	0.36	0.27	0.43	0.54	0.50	**0.56**
Table	0.34	0.41	0.53	0.55	0.45	**0.66**
Desk	0.29	0.19	0.24	**0.56**	0.36	0.52
Dresser	0.37	0.23	0.34	**0.60**	0.44	0.47
Sofa	0.46	0.28	0.35	**0.55**	0.20	0.46
Monitor	0.54	0.34	0.59	0.55	0.41	**0.69**
Night-stand	0.19	0.18	0.10	**0.59**	0.16	0.43
Toilet	0.24	0.17	0.28	0.49	0.22	**0.50**
Mean	0.34	0.24	0.52	0.56	0.35	**0.63**

The number in bold denotes it is the best result.

**Table 3:** Shape completion results ((CD $\times \;10^{3}$) and F−Score@1%) on ModelNet40 point cloud dataset (2048 points).

Methods	CD	F-Score@1%
PCN [3]	3.920	0.42
Top-Net [5]	5.974	0.32
ECG [36]	2.486	0.34
MSN [7]	2.600	0.45
Vrc-Net [9]	2.455	0.44
PCA-Net (Ours)	**2.260**	**0.54**

The number in bold denotes it is the best result.

**Table 4:** Shape completion results (CD $\times \;10^{3})$ on ShapeNet-Part point cloud dataset (2048 points).

Category	PCN [3]	Top-Net [5]	ECG [36]	MSN [7]	PCA-Net (Ours)
Airplane	1.284	2.100	1.935	0.861	**0.793**
Bag	9.570	8.596	11.109	**7.196**	8.334
Cap	20.519	18.437	**10.870**	11.243	14.960
Car	4.000	4.495	3.294	4.796	**3.174**
Chair	2.638	3.467	2.497	4.130	**1.197**
Earphone	12.932	9.505	20.211	**9.176**	9.405
Guitar	0.630	0.801	1.183	0.481	**0.349**
Knife	0.862	1.002	1.297	**0.652**	1.244
Lamp	6.823	8.571	7.626	**4.077**	5.916
Laptop	2.185	2.335	3.233	1.250	**1.119**
Motorbike	3.915	3.767	4.305	**2.587**	3.505
Mug	7.254	6.696	10.523	8.196	**4.970**
Pistol	2.326	3.203	3.374	**1.533**	2.447
Skateboard	3.491	3.998	5.500	2.275	**2.118**
Rocket	4.409	4.424	6.036	**2.486**	3.800
Table	3.324	4.621	3.677	3.586	**2.697**
Mean	2.545	3.805	1.908	2.054	**1.898**

The number in bold denotes it is the best result.

**Table 5:** Supervised shape completion results ((CD $\times \;10^{3}$) and F−Score@1%) on Modelnet10 point cloud dataset (2048 points).

Methods	CD	F-Score@1%
PCN [3]	4.334	0.34
Top-Net [5]	5.433	0.24
ECG [36]	3.988	0.22
MSN [7]	2.778	0.44
Vrc-Net [9]	2.982	0.35
PCA-Net (Ours)	**2.325**	**0.45**

The number in bold denotes it is the best result.

**Table 6:** Comparison of classification accuracy on ModelNet40 (1024 points), where PointCNN [20] classification accuracy is taken from the results of that paper.

Methods	PointCNN [20]	Our-Point	Our-CBAM	Our-Offset	Our-SE
Acc (%)	92.5	91.9	92.9	92.9	**93.1**

The number in bold denotes it is the best result.

**Table 7:** Shape completion results (CD $\times \;10^{3}$) on learning-based bilateral interpolation and learning-based trilinear interpolation.

Methods	Learning-Based Bilateral Interpolation [12]	Learning-Based Trilinear Interpolation (Ours)
CD	2.439	**2.125**

The number in bold denotes it is the best result.

## Data Availability

Publicly available datasets were analyzed in this study. This data can be found here: http://www.cvlibs.net/datasets/kitti/ (accessed on 8 July 2022).
